# Genetics and mapping of a new anthracnose resistance locus in Andean common bean Paloma

**DOI:** 10.1186/s12864-017-3685-7

**Published:** 2017-04-18

**Authors:** Sandra Aparecida de Lima Castro, Maria Celeste Gonçalves-Vidigal, Thiago Alexandre Santana Gilio, Giselly Figueiredo Lacanallo, Giseli Valentini, Vanusa da Silva Ramos Martins, Qijian Song, Marta Zulema Galván, Oscar P. Hurtado-Gonzales, Marcial Antonio Pastor-Corrales

**Affiliations:** 1 Departamento de Agronomia, Universidade Estadual de Maringá, Maringá, Paraná Brazil; 2 Soybean Genomics and Improvement Laboratory, United States Department of Agriculture, Agricultural Research Service, Beltsville, MD USA; 3 CONICET, Laboratorio de Biotecnología, Estación Experimental Agropecuaria Salta, Instituto Nacional de Tecnología Agropecuaria, Cerrillos, Salta Argentina

**Keywords:** *Phaseolus vulgaris*, *Colletotrichum lindemuthianum*, Genetic resistance, KASP markers, SNP markers

## Abstract

**Background:**

The Andean cultivar Paloma is resistant to Mesoamerican and Andean races of *Colletotrichum lindemuthianum*, the fungal pathogen that causes the destructive anthracnose disease in common bean. Remarkably, Paloma is resistant to Mesoamerican races 2047 and 3481, which are among the most virulent races of the anthracnose pathogen. Most genes conferring anthracnose resistance in common bean are overcome by these races. The genetic mapping and the relationship between the resistant *Co-Pa* gene of Paloma and previously characterized anthracnose resistance genes can be a great contribution for breeding programs.

**Results:**

The inheritance of resistance studies for Paloma was performed in F_2_ population from the cross Paloma (resistant) × Cornell 49–242 (susceptible) inoculated with race 2047, and in F_2_ and F_2:3_ generations from the cross Paloma (resistant) × PI 207262 (susceptible) inoculated with race 3481. The results of these studies demonstrated that a single dominant gene confers the resistance in Paloma. Allelism tests performed with multiple races of *C. lindemuthianum* showed that the resistance gene in Paloma, provisionally named *Co-Pa*, is independent from the anthracnose resistance genes *Co-1, Co-2, Co-3, Co-4, Co-5, Co-6, Co-12, Co-13, Co-14, Co-15* and *Co-16*. Bulk segregant analysis using the SNP chip BARCBean6K_3 positioned the approximate location of *Co-Pa* in the lower arm of chromosome Pv01. Further mapping analysis located the *Co-Pa* gene at a 390 kb region of Pv01 flanked by SNP markers SS82 and SS83 at a distance of 1.3 and 2.1 cM, respectively.

**Conclusions:**

The results presented here showed that Paloma cultivar has a new dominant gene conferring resistance to anthracnose, which is independent from those genes previously described. The linkage between the *Co-Pa* gene and the SS82 and SS83 SNP markers will be extremely important for marker-assisted introgression of the gene into elite cultivars in order to enhance resistance.

**Electronic supplementary material:**

The online version of this article (doi:10.1186/s12864-017-3685-7) contains supplementary material, which is available to authorized users.

## Background

The common bean (*Phaseolus vulgaris* L.) is a rich and inexpensive source of protein, fiber, and other important micronutrients, and a vital component for fighting hunger among millions of the poorest populations from Africa and Latin America [1, 2]. Multiple phenotypic, molecular and genomic traits distinguish the diversity of wild and domesticated common beans into two genetically differentiated gene pools known as Mesoamerican and Andean [3–5]. Mesoamerican and Andean dry beans which are commercially important throughout the world, are cultivated in the North, Central and South America, some countries of Africa and Europe [6].

Among the many diseases of common bean, anthracnose, caused by *Colletotrichum lindemuthianum* (Sacc. and Magnus) Briosi and Cavara, is one of the most widespread and severe [7, 8]. Yield reduction in susceptible cultivars can reach up to 100%, under conditions of high humidity and moderate temperatures that propitiate the pathogen’s growing. Although genetic resistance is the most cost-effective strategy to manage the anthracnose disease, the wide virulence diversity of *C. lindemuthianum* still remains the greatest challenge to develop common bean cultivars with effective and durable anthracnose resistance. Approximately 247 different virulent strains of *C. lindemuthianum*, known as races, have been described from different worldwide common bean production areas [9].

The virulence diversity of *C. lindemuthianum* falls into two distinct groups that correspond to the Andean and Mesoamerican gene pools of the common bean [10]. Mesoamerican races are virulent on both Mesoamerican and Andean common bean cultivars, however Andean races are more specific: they tend to be more virulent on common beans from the Andean pool [10–12]. Nonetheless, Andean common bean cultivars often provide effective resistance to highly virulent Mesoamerican races of anthracnose pathogen [10]. Genetic diversity exhibited by Mesoamerican common bean cultivars are greater than the one demonstrated by the Andean cultivars [13]. Thus, this is one of the reasons why Mesoamerican common bean has been used more than Andean to investigate resistance sources to anthracnose pathogen.

Previous studies have identified 19 dominant anthracnose resistance genes, which were either identified on Mesoamerican or Andean common bean gene pool. The Mesoamerican genes include: *Co-2*, *Co-3* (and its alleles *Co-3* ^*2*^, *Co-3* ^*3*^, *Co-3* ^*4*^, *Co-3* ^*5*^), *Co-4* (and it alleles *Co-4* ^*2*^, *Co-4* ^*3*^), *Co-5* (and its allele *Co-5* ^*2*^), *Co-6*, *Co-11*, Co*-16*, *Co-17*, *Co-u*, and *Co-v* [8, 14–26]. The Andean genes include: *Co-1* (and its alleles *Co-1* ^*2*^, *Co-1* ^*3*^, *Co-1* ^*4*^, *Co-1* ^*5*^), *Co-12*, *Co-13*, *Co-14*, *Co-15*, *Co-x*, *Co-w*, *Co-y*, and *Co-z* [26–33].

The anthracnose resistance genes have been mapped on seven of the 11 chromosomes of common bean [8, 22–24, 30, 34]. Several of these genes have been reported on linkage groups Pv01, Pv04 and Pv11 that contain gene clusters conferring resistance to anthracnose and other diseases [8]. For example, linkage group Pv01 contains a resistance cluster to anthracnose (*Co-1*, *Co-14*, *Co-x* and *Co-w*), rust (*Ur-9*), and angular leaf spot (*Phg-1*) [8, 21].

The search for new sources of Andean resistance to *C. lindemuthianum* is extremely important for common bean breeding programs in tropical and subtropical regions. Paloma is a white-seeded highly productive Andean bean with desirable agronomic characteristics [35]. Therefore, this cultivar has been recommended by the Instituto Nacional de Tecnologia Agropecuaria (INTA), Salta, Argentina for cultivation and became popular for human consumption in Europe, especially in Spain and Italy [35]. Experiments conducted in Brazil revealed that Paloma is resistant to Andean and Mesoamerican races of *C. lindemuthianum*.

Given the importance to identify new sources of anthracnose resistance in Andean common bean, the objectives of this study were: 1) to characterize the genetic resistance of Andean common bean cultivar Paloma to *C. lindemuthianum*, 2) to map the anthracnose resistance gene of cultivar Paloma and 3) to identify molecular markers linked to this resistance gene.

## Methods

### Plant materials and phenotypic reaction to *Colletotrichum lindemuthianum*

The cultivar Paloma and other Andean common bean cultivars were inoculated with Andean races 2, 7, 19, 23, and 55 and with Mesoamerican races 65, 73, 89, 449, 453, 1545, 2047 and 3481 of *C. lindemuthianum*. The other Andean bean cultivars, with their respective anthracnose resistance genes, included in this study were: Michigan Dark Red Kidney (MDRK) (*Co-1*), Kaboon (*Co-1* ^*2*^), Perry Marrow (PM) (*Co-1* ^*3*^), AND 277 (*Co-1* ^*4*^), Widusa (*Co-1* ^*5*^), Jalo Vermelho (JV) (*Co-12*), Jalo Listras Pretas (JLP) (*Co-13*), Pitanga (*Co-14*), Corinthiano (*Co-15*), and Jalo EEP 558 (*Co-w, Co-x, Co-y and Co-z*). Inoculation of *C. lindemuthianum* races was conducted on 10 plants of each cultivar, when the first trifoliate leaf was fully developed (stage V_3_). Inoculum for each race was obtained from young bean-pod medium and placed in test tubes containing 5 mL of water-agar, which were kept upright at 22 °C for 14 days according to Cárdenas et al. [36]. After 14 days, a spore suspension was prepared with sterile distilled water containing Tween 20 and adjusted to a concentration of 1.2 × 10^6^ spores mL^−1^. Seedlings with fully developed first trifoliate leaves of common bean cultivars parents, F_2_, and F_2:3_ were inoculated with spores of selected races of *C. lindemuthianum* as described by Cárdenas et al. [36], using an electric air compressor De Vilbiss number 15. After inoculation, the plants were maintained at >95% relative humidity at 20 ± 2 °C with controlled luminosity (12 h light of 680 lx/12 h dark) in a mist chamber for 3 days. After this period, the plants were removed from the mist chamber and transferred to benches with suitable environment at 22 °C with artificial light (12 h day length at 25 °C) for 7 days. Anthracnose disease reactions were rated visually using a scale from 1 to 9, as previously described [37]. Plants with scores from 1 to 3 were considered resistant while those with scores from 4 to 9 were considered susceptible.

### Inheritance test

In order to determine the resistance inheritance in cultivar Paloma, plants from the F_2_ populations and parental cultivars were grown under greenhouse conditions. Inoculations with *C. lindemuthianum* races were conducted when the first trifoliate leaf was fully developed (stage V_3_). Races 2047 and 3481 were chosen for the inoculations due to the contrasting pathogenic spectrum observed in the parents from the crosses Paloma (resistant) × PI 207262 (susceptible) and Paloma (resistant) × Cornell 49–242 (susceptible). Moreover, the races 2047 and 3481 showed high virulence index overcoming the resistance, respectively, in eleven and seven differential cultivars.

Ninety-nine F_2_ plants derived from the cross Paloma (resistant) × Cornell 49–242 (susceptible) and 10 plants from each parent were inoculated with race 2047. Additionally, another set of 90 F_2_ plants derived from the cross Paloma (resistant) × PI 207262 (susceptible) were tested with race 3481. Similarly, 73 F_2:3_ families derived from the cross Paloma (resistant) × PI 207262 (susceptible) were inoculated with race 3481. The anthracnose phenotype obtained from these F_2:3_ families was used to compare the phenotype of their corresponding F_2_ plants. These populations were produced at the Núcleo de Pesquisa Aplicada à Agricultura (Nupagri) of the Universidade Estadual de Maringá (UEM), Maringá, Paraná, Brazil.

The anthracnose virulence phenotype of races 2047 and 3481 were confirmed by inoculating the set of 12 common bean anthracnose differential cultivars with each of these races [38], because they are used to identify *C. lindemuthianum* races. Race 2047 was virulent on all differential cultivars, except on G 2333. Further, race 3481 was virulent on seven of the 12 differentials, including G 2333 the most resistant genotype among the 12 differential cultivars [39].

### Allelism tests

Allelism tests were carried out with 16 different F_2_ populations with the aim to investigate the independence of the anthracnose resistance gene in Paloma from those previously characterized resistance genes in common bean. For that, Paloma was crossed with the following cultivars: Michigan Dark Red Kidney (MDRK) (*Co-1*), Cornell 49–242 (*Co-2*), Ouro Negro (*Co-3* ^*4*^), TO (*Co-4*), G 2333 (*Co-4* ^*2*^ *)*, PI 207262 (*Co-4* ^*3*^), TU (*Co-5*), AB 136 (*Co-6*), Jalo Vermelho (*Co-12*), Jalo Listras Pretas (*Co-13*), Pitanga (*Co-14*), Corinthiano (*Co-15*), Crioulo 159 (*Co-16*), Amendoim Cavalo, Perla, and Jalo Pintado 2. Paloma was used as the female parent. F_1_ seeds from the crosses mentioned above were grown in pots and the dominant morphological markers, such as pink or purple flower color and indeterminate growth habit in male parents, were verified to confirm hybridization. Variable numbers of F_2_ seeds from each cross, depending on seed availability, were inoculated with the races 65, 73 and 2047. Spore increase, inoculum preparation, inoculations and evaluations of the anthracnose phenotypes were performed in the same manner as described in the first section of Methods. To test the hypothesis of independence of the resistance gene in Paloma, the races 65, 73 and 2047 were carefully chosen based on R × R reaction produced in the parents. It is important to mention that races 73 and 65 are the most frequent races in common bean producing areas in Brazil, being present in 13 and 12 out of the 14 states monitored, respectively [9].

### Bulk segregant analysis and SNP assay

Plant tissue from Paloma, PI 207262 and from 73 F_2_ plants, derived from the cross Paloma × PI 207262, were collected for total DNA extraction with the PureLink Genomic Plant DNA Purification Kit, following the manufacturer’s instructions. DNA bulks were used to perform a bulk segregant analysis to identify SNP markers potentially linked to the resistance gene in Paloma [40]. Briefly, two contrasting DNA bulks were constructed by pooling equal volumes of fluorometrically standardized DNA from six F_2_ plants, which were homozygous for the resistant genotype (resistant bulk, RB) and homozygous for the susceptible genotype (susceptible bulk, SB). Homozygous resistant and susceptible F_2_ plants were identified based on the phenotypic assessment evaluation conducted on the F_2:3_ families of the same cross inoculated with *C. lindemuthianum* race 3481.

The bulk segregant analysis with SNP markers was performed at the Soybean Genomics and Improvement Laboratory of the USDA-ARS at the Beltsville Agricultural Research Center, Maryland, USA, using the Illumina BARCBean6K_3 BeadChip, containing 5,399 Single Nucleotide Polymorphism (SNP) DNA markers [41]. SNP genotyping with Illumina BeadChip was carried out as described by Song et al. [42] and performed according to the Infinium HD Assay Ultra Protocol (Illumina, Inc. San Diego, CA). The BeadChip was imaged using the Illumina BeadArray Reader to measure fluorescence intensity. For each SNP locus, alleles were automatically called with the GenomeStudio Genotyping Module v1.8.4 (Illumina, San Diego, CA) and later manually checked. A SNP was considered positive when polymorphism between Paloma (resistant) and PI 207262 (susceptible) was observed and the SB clustered with PI 207262, and the RB clustered with cultivar Paloma.

Positively associated SNP markers derived from BARCBean6K_3 BeadChip were selected to develop Kompetitive Allele Specific PCR (KASP) markers. KASP primer sequences were designed using the software PrimerExpress. KASP assays were performed according to the manufacturer’s instructions. Briefly, PCR reaction was conducted in a 10 μL final volume containing 5 μL of 2X premade KASP master mix (LGC, Middlesex, UK), 0.14 μL of primer mix (Sigma-Aldrich, St. Louis, USA) and 20–40 ng of genomic DNA. After PCR amplification, PCR plates were scanned in a Mx3000P qPCR Reader and allele scores were obtained using the MxPro qPCR software (Agilent, St. Clara, CA).

### Statistical analyses

Segregation analyses of the disease reaction of 99 F_2_ individuals from the cross Paloma (resistant) × Cornell 49–242 (susceptible) and 90 F_2_ plants from the cross Paloma (resistant) × PI 207262 (susceptible), inoculated respectively with races 2047 and 3481, were performed with the chi-square (*χ* ^2^) test using the Genes software [43]. These analyses followed the Mendelian segregation hypothesis of 3 R (resistant) to 1 rr (susceptible). The segregation analyses of the F_2:3_ families were performed using the chi-square goodness-of-fit test (*χ* ^2^) for the hypothesis of Mendelian segregation 1:2:1 (RR:RS:SS). The segregation analysis of the disease reaction of F_2_ populations from each cross (R × R) were conducted using the chi-square test of goodness-of-fit to the hypothesis of Mendelian segregation 15:1 (R:S) for two independent genes.

The SNP markers were used to map the resistance gene in Paloma using DNA of the F_2_ plants from Paloma × PI 207262 cross. Genetic distances between the SNP markers and the new anthracnose resistance gene in Paloma was estimated using software JoinMap 4.0 [44]. Default settings of Regression Mapping algorithm based on the Kosambi map function were attributed to define the linkage order and distances in centimorgans (cM). A minimum likelihood of odds (LOD) ≥3.0 and a maximum distance of ≤50 cM were used to test linkages among markers. Genetic linkage map, containing the genetic distances between SNP markers and the anthracnose resistance gene in Paloma, was drawn using the software MapChart [45]. The SNP markers flanking the anthracnose resistance locus in Paloma were used to determine the physical region of the new locus. Candidate annotated genes in the region were identified by inspecting the genome browser from the reference genome of common bean in phytozome.org [5].

## Results

### Phenotypic reaction of cultivar Paloma and other Andean cultivars

Table 1 shows the disease reaction of Paloma and the other anthracnose resistance Andean cultivars, when inoculated with *C. lindemuthianum* Andean races (2, 7, 19, 23 and 55) and Mesoamerican races (65, 73, 89, 449, 453, 1545, 2047 and 3481). Paloma was resistant to Andean races 23 and 55, as well as to Mesoamerican races 65, 73, 1545, 2047 and 3481.

**Table 1:** Reaction of Andean common bean cultivars to various races of *Colletotrichum lindemuthianum*

Cultivar	Gene	Linkage group	Reaction to *C. lindemuthianum* races												
			2	7	19	23	55	65	73	89	449	453	1545	2047	3481
Paloma	*Co-Pa*	Pv01	S^e^	S	S	R	R	R	R	S	S	S	R	R	R
MDRK^a^	*Co-1*	Pv01	S	S	S	S	S	R	R	R	R	R	R	S	R
Kaboon	*Co-1* ^*2*^	Pv01	R^f^	R	R	R	S	R	R	R	R	R	R	S	R
PM^b^	*Co-1* ^*3*^	Pv01	R	S	R	S	S	R	R	R	R	S	R	S	R
AND 277	*Co-1* ^*4*^	Pv01	NA^g^	NA	S	R	R	R	R	R	NA	R	R	R	R
Widusa	*Co-1* ^*5*^	Pv01	R	R	S	S	S	R	R	S	R	R	R	S	S
JV^c^	*Co-12*	-	NA	S	S	R	R	R	S	R	R	R	R	S	S
JLP^d^	*Co-13*	Pv03	NA	S	S	S	S	R	R	R	S	S	R	S	R
Pitanga	*Co-14*	Pv01	NA	NA	R	R	R	R	R	S	NA	S	S	R	S
Corinthiano	*Co-15*	Pv04	R	NA	S	R	S	R	R	R	R	S	R	R	S
Jalo EEP558	*Co-w* *Co-x* *Co-y* *Co-z*	Pv01 Pv04	S	S	S	NA	S	R	R	S	R	R	R	S	NA

^a^Michigan Dark Red Kidney; ^b^Perry Marrow; ^c^Jalo Vermelho; ^d^Jalo Listras Pretas; ^e^Susceptible; ^f^Resistant; ^g^Not Available

### Inheritance of anthracnose resistance in Paloma

The inheritance of resistance study conducted with 99 F_2_ plants from the cross between Paloma (resistant) × Cornell 49–242 (susceptible), inoculated with *C. lindemuthianum* race 2047, exhibited a segregation pattern fitted in a 3R:1S ratio (*χ* ^2^ = 0.084; *p* = 0.77), showing that resistance in Paloma to race 2047 is conferred by a dominant gene. The inheritance of resistance of 90 F_2_ plants from the Paloma (resistant) × PI 207262 (susceptible) cross, inoculated with race 3481, resulted in a segregation that fitted a ratio of 3R:1S. Similarly, the segregation of the 73 F_2:3_ families from the Paloma (resistant) × PI 207262 (susceptible) cross, inoculated with race 3481, fitted a ratio of 1RR:2RS:1SS. This fact suggested that a single dominant gene in Paloma confers resistance to race 3481 of *C. lindemuthianum* (Table 2).

**Table 2:** Inheritance of anthracnose resistance in the common bean cultivar Paloma

Crosses	Generation	Observed ratio	Expected ratio	*χ* ^2^	*P*-value
Race 2047 of *Colletotrichum lindemuthianum*					
Paloma	RP^a^	10:0			
Cornell 49–242	SP^b^	0:10			
Paloma × Cornell 49–242	F_2_	73:26	74.25R^c^:24.75S^d^	0.084	0.77
Race 3481 of *Colletotrichum lindemuthianum*					
Paloma	RP	10:0			
PI 207262	SP	0:10			
Paloma × PI 207262	F_2_	65:25	67.50R:22.50S	0.370	0.54
Paloma × PI 207262	F_2:3_	12:43:18	18.25RR:36.52RS:18.25SS	3.301	0.19

^a^Resistant Parent; ^b^Susceptible Parent; ^c^Resistant; ^d^Susceptible

### Allelism tests

Genetic analysis of F_2_ populations involving parental cultivars with resistant reactions to various races of the anthracnose pathogen showed that the resistance gene in Paloma is independent from previously characterized anthracnose resistance genes (Table 3). The allelism tests for the F_2_ populations derived from crosses between cultivar Paloma with the common bean cultivars MDRK, Cornell 49–242, Ouro Negro, TO, G 2333, PI 207262, TU, and AB 136 showed a 15:1 segregation ratio in each population, indicating that two dominant genes conferred resistance to anthracnose.

**Table 3:** Allelism tests for the anthracnose resistance gene in the common bean cultivar Paloma

Crosses	Resistance Gene	Race	Linkage Group	Observed Ratio		Expected Ratio (R:S)	*χ* ^2^	*P*-value
				R^d^	S^e^			
Paloma × MDRK^a^	*Co-1*	73	Pv01	92	8	15:1	0.523	0.47
Paloma × Cornell 49–242	*Co-2*	65	Pv11	84	6	15:1	0.027	0.87
Paloma × Ouro Negro	*Co-3* ^*4*^	73	Pv04	118	9	15:1	0.152	0.70
Paloma × TO	*Co-4*	65	Pv08	111	8	15:1	0.045	0.83
Paloma × G 2333	*Co-4* ^*2*^	2047	Pv08	155	13	15:1	0.635	0.43
Paloma × PI 207262	*Co-4* ^*3*^	65	Pv08	137	10	15:1	0.076	0.78
Paloma × TU	*Co-5*	65	Pv07	109	6	15:1	0.209	0.65
Paloma × AB 136	*Co-6*	65	Pv07	106	8	15:1	0.114	0.73
Paloma × Jalo Vermelho	*Co-12*	65	-	107	7	15:1	0.002	0.96
Paloma × JLP^b^	*Co-13*	65	Pv03	110	8	15:1	0.056	0.81
Paloma × Pitanga	*Co-14*	73	Pv01	56	4	15:1	0.018	0.89
Paloma × Corinthiano	*Co-15*	2047	Pv04	94	6	15:1	0.011	0.92
Paloma × Crioulo 159	*Co-16*	2047	Pv04	94	6	15:1	0.011	0.92
Paloma × Perla	*-*	65	-	111	19	15:1	0.320	0.57
Paloma × AC^c^	*-*	2047	-	109	7	15:1	0.009	0.92
Paloma × Jalo Pintado 2	*-*	2047	-	90	6	15:1	0.001	0.99

^a^Michigan Dark Red Kidney; ^b^Jalo Listras Pretas; ^c^Amendoim Cavalo; ^d^Resistant; ^e^Susceptible

Segregations observed in the F_2_ populations of crosses of Paloma with the cultivars Jalo Vermelho (*p* = 0.96), Jalo Listras Pretas (*p* = 0.81), Pitanga (*p* = 0.89), Corinthiano (*p* = 0.92), Crioulo 159 (*p* = 0.92), Perla (*p* = 0.57), Amendoim Cavalo (*p* = 0.92), and Jalo Pintado 2 (*p* = 0.99) also exhibited a 15R:1S ratio. These results demonstrated the independence of the dominant gene present in Paloma from previously characterized anthracnose resistance genes shown on Table 3. Thus, the gene in Paloma is independent from *Co-1*, *Co-2*, *Co-3* ^*4*^, *Co-4, Co-4* ^*2*^ *, Co-4* ^*3*^ *, Co-5, Co-6, Co-12, Co-13, Co-14, Co-15* and *Co-16,* as well as from unnamed resistance genes in common bean cultivars Perla, Amendoim Cavalo and Jalo Pintado 2.

### BSA and SNP genotyping using BARCBEAN6K_3 BeadChip

Table 4 displays the 33 genotyped SNPs on the resistant and susceptible bulks constructed using DNA of the F_2_ individuals derived from the Paloma × PI 207262 cross. These SNPs were positively associated with the new Andean gene *Co-Pa* present in Paloma. All positive SNP markers were positioned spanning a 2.779 Mb region of the lower arm of chromosome Pv01. The SNPs were positioned in a region between 60.170 and 75.989 cM on the common bean chromosome Pv01, using the linkage map presented by Song et al. [41]. The region containing *Co-Pa* was flanked by SNP markers ss715645931 (48,838,736 bp) and ss715645293 (51,617,802 bp).

**Table 4:** SNP markers associated with the resistant gene in Paloma discovered by BSA

BARCBEAN6K_3 SNP ID	NCBI ss# id	Genetic distance (cM)^a^	Physical position (bp)
BARCPV_1.0_Chr01_48838736_G_A	ss715645931	60.170	48,838,736
BARCPV_1.0_Chr01_48865391_A_G	ss715645929	60.170	48,865,391
BARCPV_1.0_Chr01_48925715_G_A	ss715645922	60.767	48,925,715
BARCPV_1.0_Chr01_48985087_G_A	ss715645915	60.963	48,985,087
BARCPV_1.0_Chr01_48993566_T_C	ss715645914	60.963	48,993,566
BARCPV_1.0_Chr01_49001931_C_T	ss715645913	60.963	49,001,931
BARCPV_1.0_Chr01_49030363_T_G	ss715645911	60.963	49,030,363
BARCPV_1.0_Chr01_49102396_T_C	ss715645906	-	49,102,396
BARCPV_1.0_Chr01_49112768_C_A	ss715645904	61.543	49,112,768
BARCPV_1.0_Chr01_49123029_C_T	ss715645903	-	49,123,029
BARCPV_1.0_Chr01_49181319_T_C	ss715645900	61.920	49,181,319
BARCPV_1.0_Chr01_49192664_G_A	ss715645899	62.445	49,192,664
BARCPV_1.0_Chr01_49261257_G_A	ss715645892	62.034	49,261,257
BARCPV_1.0_Chr01_49329790_T_C	ss715645883	62.834	49,329,790
BARCPV_1.0_Chr01_49361978_A_C	ss715645881	62.834	49,361,978
BARCPV_1.0_Chr01_49487812_C_T	ss715645868	63.341	49,487,812
BARCPV_1.0_Chr01_49517944_C_T	ss715645866	63.341	49,517,944
BARCPV_1.0_Chr01_49531622_G_A	ss715645864	63.341	49,531,622
BARCPV_1.0_Chr01_49546017_T_C	ss715645861	63.341	49,546,017
BARCPV_1.0_Chr01_49588715_A_G	ss715645859	63.933	49,588,715
BARCPV_1.0_Chr01_49606339_T_C	ss715645857	64.862	49,606,339
BARCPV_1.0_Chr01_49617274_G_A	ss715645856	63.933	49,617,274
BARCPV_1.0_Chr01_49637944_C_A	ss715645853	63.933	49,637,944
BARCPV_1.0_Chr01_49657760_C_T	ss715645852	63.933	49,657,760
BARCPV_1.0_Chr01_49671031_G_A	ss715645935	64.122	49,671,031
BARCPV_1.0_Chr01_49678615_G_A	ss715645927	64.122	49,678,615
BARCPV_1.0_Chr01_49694647_G_A	ss715645910	64.493	49,694,647
BARCPV_1.0_Chr01_49742126_G_A	ss715645862	64.648	49,742,126
BARCPV_1.0_Chr01_49749711_T_C	ss715645855	64.648	49,749,711
BARCPV_1.0_Chr01_49841858_G_A	ss715645286	65.168	49,841,858
BARCPV_1.0_Chr01_50155987_T_C	ss715645258	66.999	50,155,987
BARCPV_1.0_Chr01_50203547_C_T	ss715645254	67.201	50,203,547
BARCPV_1.0_Chr01_50546985_T_C	ss715645248	-	50,54,6985
BARCPV_1.0_Chr01_51289521_C_A	ss715645302	75.989	51,289,521
BARCPV_1.0_Chr01_51353193_C_T	ss715645299	-	51,353,193
BARCPV_1.0_Chr01_51617802_G_A	ss715645293	-	51,61,7802

^a^Linkage position (cM) on Pv01 of markers in the Stampede × Red Hawk (S × R) F_2_ population [41]

### KASP assay development and gene mapping

**Fig. 1:**
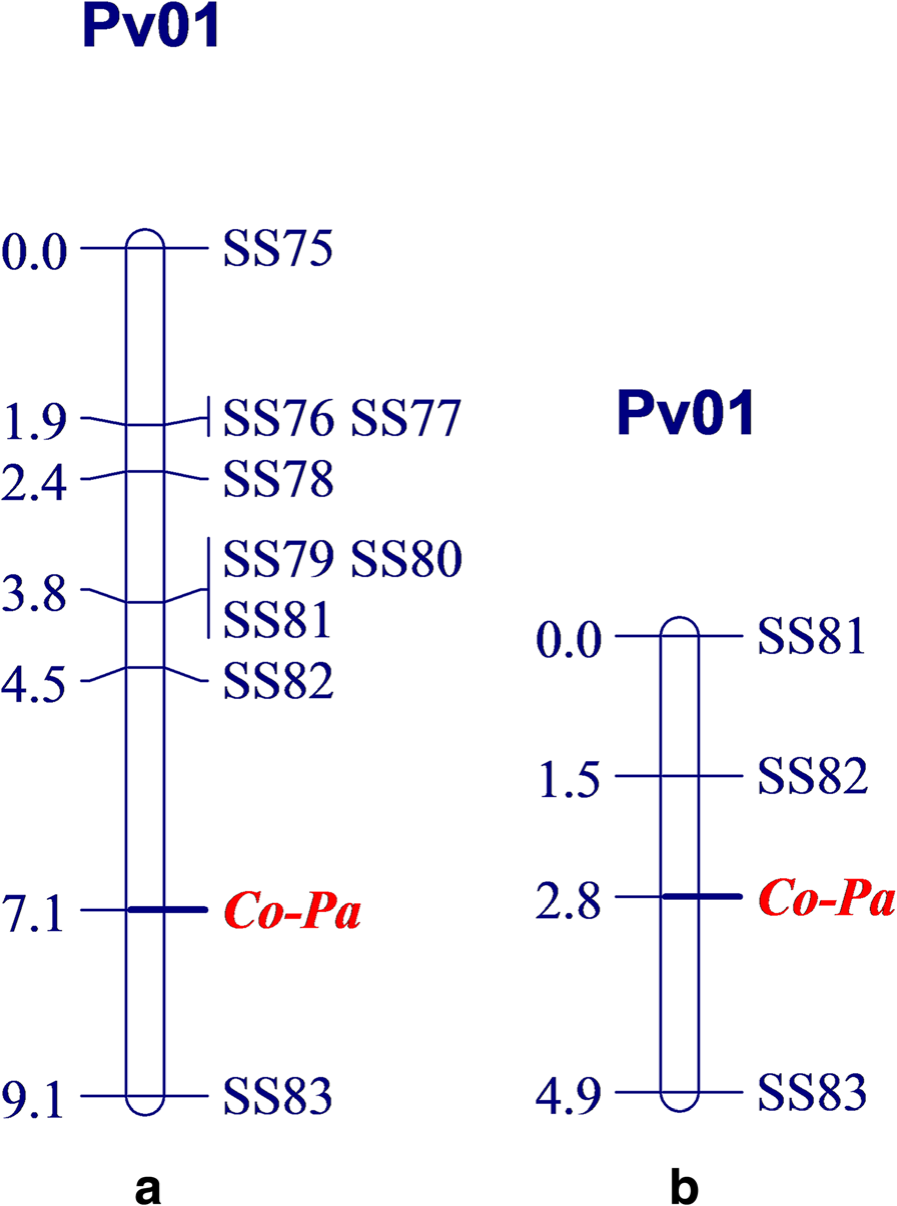
Genetic linkage map of the SNP markers and anthracnose resistance gene in Paloma. **a** Linkage group Pv01 for the *Co-Pa* in Paloma and nine SNP markers genotyped in 73 F_2_ plants of Paloma × PI 207262; **b** Linkage group Pv01 for the *Co-Pa* and three close linked SNP markers genotyped in 163 F_2_ individuals of Paloma × PI 207262

Nine positive SNPs, distributed in the candidate physical region, were selected for the development of KASP assays to genotype the 73 F_2_ plants from the cross Paloma × PI 207262 (Table 5). The phenotypic evaluations carried out in the corresponding F_2:3_ families were combined with the SNP genotyping of the F_2_ population. A total of six F_2_ plants revealed breakpoints, suggesting that the Andean anthracnose resistance gene in Paloma is flanked by markers SS82 (50,546,985 bp) and SS83 (50,155,987 bp), spanning a 390,998 bp region of chromosome Pv01 (Additional file 1: Table S1). The mapping analysis of these SNP markers associated with the 73 F_2_ plants of Paloma × PI 207262 cross demonstrated that the resistance locus to *C. lindemuthianum* of Paloma is located between the markers SS82 and SS83 at a distance of 2.6 and 2.0 cM, respectively (Fig. 1a).

**Table 5:** KASP primers associated with the resistant gene in Paloma

SNP id	NCBI ss# id	Physical position (bp)^a^	BARCBEAN6K_3 SNP ID	Primers sequences^b, c, d^
SS75	ss715645931	48838736	BARCPV_1.0_Chr01_48838736_G_A	F1: GAAGGTGACCAAGTTCATGCTGTGTTATGTGTAGATATATTGAAAGGC F2: GAAGGTCGGAGTCAACGGATTACTGTGTTATGTGTAGATATATTGAAAGGT R: TGATCTCTTAATGGGAGGTTTGTTGCTT
SS76	ss715645913	49001931	BARCPV_1.0_Chr01_49001931_C_T	F1: GAAGGTGACCAAGTTCATGCTCAGCTTCTACCCGTGGCTGC F2: GAAGGTCGGAGTCAACGGATTGCAGCTTCTACCCGTGGCTGT R: GTCGCTTCTAGTTGTCTTTAATTATCTTAT
SS77	ss715645900	49181319	BARCPV_1.0_Chr01_49181319_T_C	F1: GAAGGTGACCAAGTTCATGCTCAGTTGAACATTATGTTACAGACTACTTT F2: GAAGGTCGGAGTCAACGGATTCAGTTGAACATTATGTTACAGACTACTTC R: AAACCTAAACTAATAACAAAATTTATGTAT
SS78	ss715645883	49329790	BARCPV_1.0_Chr01_49329790_T_C	F1: GAAGGTGACCAAGTTCATGCTATTAGGCTGTTCAAGCTTTCCAGGT F2: GAAGGTCGGAGTCAACGGATTAGGCTGTTCAAGCTTTCCAGGC R: CACCCCAATTACATTTTCTCTTTATGAATT
SS79	ss715645868	49487812	BARCPV_1.0_Chr01_49487812_C_T	F1: GAAGGTGACCAAGTTCATGCTGCCGCTGGTGAAGATGAATACC F2: GAAGGTCGGAGTCAACGGATTCGCCGCTGGTGAAGATGAATACT R: CGTGTATGCGACCCGGTCGAA
SS80	ss715645856	49617274	BARCPV_1.0_Chr01_49617274_G_A	F1: GAAGGTGACCAAGTTCATGCTATATTTGTTATTATGGTGTCATCATATCTTAA F2: GAAGGTCGGAGTCAACGGATTATATTTGTTATTATGGTGTCATCATATCTTAAT R: GTTTGAACTGATGTAATAACTGAACCTATA
SS81	ss715645862	49742126	BARCPV_1.0_Chr01_49742126_G_A	F1: GAAGGTGACCAAGTTCATGCTATTGAAGATAAGTACTATCGATGATATCAG F2: GAAGGTCGGAGTCAACGGATTGTATTGAAGATAAGTACTATCGATGATATCAA R: GTGAGGTAGTTCGATCCTCTTGAGAT
SS82	ss715645258	50155987	BARCPV_1.0_Chr01_50155987_T_C	F1: GAAGGTGACCAAGTTCATGCTAAAACCAGGTTTATTGTCCTAGTTTACAA F2: GAAGGTCGGAGTCAACGGATTAACCAGGTTTATTGTCCTAGTTTACAG R: GTATCTCATCTTGTTGCAAGAGTGAATATA
SS83	ss715645248	50546985	BARCPV_1.0_Chr01_50546985_T_C	F1: GAAGGTGACCAAGTTCATGCTCATATACTTTCTGGGTGTAAACTCTGA F2: GAAGGTCGGAGTCAACGGATTATATACTTTCTGGGTGTAAACTCTGG R: CCCAGGTGTTCTGGTCATGAGTTAT

^a^Physical position of SNP in the common bean reference genome v1.0; ^b^F1: Forward 1; ^c^F2: Forward 2, ^d^R: Reverse

**Fig. 2:**
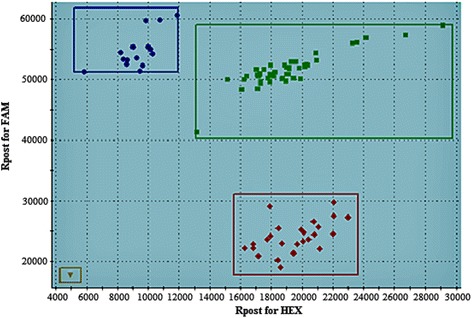
A SNP marker display. Clustering of the 73 F_2_ individuals from Paloma × PI 207262 cross: Paloma allele (*blue*), PI 207262 allele (*red*) and heterozygous allele (*green*) for the SS83 SNP marker

In order to obtain more experimental precision, the three SNP markers, which are very close linked to *Co-Pa*, were carefully chosen for genotyping the 163 F_2_ plants from Paloma × PI 207262 cross, involving the same 73 F_2_ individuals previously genotyped and a new set of 90 F_2_ plants (Additional file 2: Table S2). For that, all these plants had their phenotypic reaction evaluated using the race 3481 of the *C. lindemuthianum*. These results revealed that the anthracnose resistance gene *Co-Pa* of Paloma is located between SS82 and SS83 markers at the distance of 1.3 and 2.1 cM, respectively. Additionally, the SNP marker SS83 effectively differentiated homozygous resistant, homozygous susceptible and heterozygous individuals (Fig. 2).

### The *Co-Pa* gene of Paloma mapping to a cluster of disease resistance genes

Based on the gene annotation for the *P. vulgaris* genome V1.0 (phytozome.org), the ~390 Kbp genomic region, containing the anthracnose resistance locus from Paloma, has 46 annotated genes. Most of these genes encode proteins with predicted functions unrelated to plant pathogen defense. However, nine genes contain domains with known function related to pathogen resistance response in plants [5]. These genes are: Phvul.001G243100, Phvul.001G243200 and Phvul.001G243300 encoding phosphoinositide-specific phospholipase-C (PI-PLC) proteins. Genes Phvul.001G243500, Phvul.001G243600, Phvul.001G243700, Phvul.001G243800 and Phvul.001G245100 encode serine threonine protein kinase (STK), while Phvul.001G245300 encodes leucine-rich repeat (LRR) with a protein tyrosine kinase (PTK) domain.

## Discussion

A broad genetic base is essential for the protection of crops, especially for their protection against pathogens with high virulence diversity. The utilization of Andean common bean for the genetic improvement of the common bean crop has lagged far behind the use of Mesoamerican common bean [6]. Paradoxically, the underutilized genetic diversity of Andean common bean contains genes conferring effective resistance to highly virulent Mesoamerican races of the pathogens that cause anthracnose, angular leaf spot, and rust diseases of common bean [10, 37, 46]. Incorporating Andean anthracnose resistance genes, such as the new gene (*Co-Pa*) in Paloma in common bean-breeding programs, would make possible the development of common bean cultivars with broad resistance to *C. lindemuthianum*.

The results of this study showed that the Andean cultivar Paloma is a very important source of anthracnose resistance, particularly for the protection against Mesoamerican races of this pathogen. We observed that Paloma was resistant to *C. lindemuthianum* Andean races 23 and 55 and to Mesoamerican races 65, 73, 1545, 2047 and 3481*.* Interestingly, Paloma as shown to be effective against Mesoamerican races of the anthracnose pathogen that are frequent in countries such as: United States, Brazil and Central America. The resistance of Paloma to races 23, 55, 65 and 73 has a great importance for common bean breeding programs in Brazil, especially on places where these races are widespread [47]. Race 73 is present in 13 of the 14 states of Brazil, while race 65 is present in 12 states of this country [9]. Race 65 has also been reported in Honduras, Mexico, USA, Costa Rica, Guatemala, Puerto Rico, India and Canada [9]. Among the resistance genes in Andean cultivars, *Co-1* ^*4*^ *, Co-12* and *Co-14* are resistant to Andean race 55. This race overcomes the resistance of the Andean *Co-1, Co-1* ^*2*^, *Co-1* ^*3*^, *Co-1* ^*5*^, *Co-13* and *Co-15* resistant genes. Race 55 also overcomes the resistance of Andean common bean Jalo EEP558, which contains the *Co-w*, *Co-x*, *Co-y* and *Co-z* anthracnose resistant genes.

Paloma is also resistant to the highly virulent Mesoamerican races 2047 and 3481. Race 2047 is virulent on 11 of the 12 differential cultivars. Thus, this race overcomes the resistance of the *Co-1* and four of its alleles, *Co-2*, *Co-3*, some of the alleles of *Co-4*, *Co-5*, *Co-6*, *Co-11*, *Co-12*, *Co-13*, and the four anthracnose genes present in Jalo EEP 558. G 2333 was the only differential cultivar that was resistant to race 2047. Among the Andean anthracnose resistance genes, only *Co-14, Co-15* and C*o-1* ^*4*^ were resistant to race 2047. Similarly, race 3481 overcomes the resistance of seven of the 12 differential cultivars, including G 2333 that contains the *Co-3* ^*5*^ *, Co-4* ^*2*^ and *Co-5* ^*2*^ alleles. Andean alleles *Co-1*, *Co-1* ^*2*^, *Co-1* ^*3*^, *Co-1* ^*4*^, and gene *Co-13* were resistant to race 3481. Therefore, only AND 277 (*Co-1* ^*4*^) and Paloma (*Co-Pa*) were resistant to the highly virulent 2047 and 3481 races of *C. lindemuthianum*.

The inheritance of resistance evaluation from the cross Paloma (R) × Cornell 49–242 (S) - inoculated with race 2047, and the cross Paloma (R) × PI 207262 (S) - inoculated with race 3481, revealed the presence of a single dominant gene in Paloma. Furthermore, bulk segregant analysis, using the BARCBean6K_3 BeadChip in common bean, positioned the resistance gene in Paloma (*Co-Pa*) in the lower arm of chromosome Pv01 where other anthracnose resistant genes have been mapped. These genes include *Co-1* and its alleles, *Co-14*, *Co-x* and *Co-w*. The allelism tests showed that *Co-Pa* is independent from the allelic series *Co-1* and the *Co-14* genes*.* No allelism test was conducted with Jalo EEP558, which contains two anthracnose resistant genes (*Co-x* and *Co-w*) in Pv01. However, the anthracnose resistant phenotype of Paloma and Jalo EEP558 were different for races 55, 449, 453, and 2047, suggesting that *Co-Pa* may be different from *Co-x* and *Co-w* (Table 1).

The *Co-Pa* anthracnose resistant gene in Paloma is mapped to a cluster containing anthracnose (*Co-1* and its alleles, *Co-14*, *Co-x*, *Co-w*), rust (*Ur-9*) and angular leaf spot (*Phg-1*) disease resistant genes [21, 48–50]. Dense clusters of tightly linked resistance-associated genes in the common bean genome encode mostly NB-LRR (nucleotide-binding, leucine-rich repeat) genes [5]. The identification of this protein class across diverse plant species demonstrated that NB-LRR genes are a pillar of plant defense [51]. NB-LRR gene clusters enriched with an N-terminal coiled-coil domain (CNL) or an N-terminal *Toll*-interleukin-1 receptor (TIR)-like domain (TNL) were identified at the ends of common bean chromosomes, such as the distal part of Pv01 [5, 23]. It is worth noting that all resistance genes currently mapped on chromosome Pv01 are from common bean of Andean origin. This is a unique situation among the chromosomes of common bean containing disease resistance genes.

The combined results of SS82 and SS83 SNP markers, with the monogenic inheritance and allelism tests, confirmed the hypothesis that a single dominant gene confers resistance to *C. lindemuthianum* races 65, 73, 2047 and 3481 in the Andean common bean cultivar Paloma. Likewise, this gene is independent from those common bean anthracnose resistance genes previously characterized. The authors propose that this single dominant anthracnose resistance gene in Paloma to be temporarily named as *Co-Pa*, until a new designation is defined by the Genetics Committee of the Bean Improvement Cooperative.

Future efforts will focus on fine mapping of the 390,998 bp region in Pv01 containing the *Co-Pa*, the resistance gene in Paloma. *Co-x* and *Co-1* ^*4*^ anthracnose resistance genes have been closely mapped to *Co-Pa*. Thus, genetic analysis of segregating populations from the cross between Paloma and cultivars containing *Co-1* ^*4*^ and *Co-x* will be the subject of future studies.

The *Co-Pa* anthracnose resistant gene in Paloma provides resistance to the Mesoamerican races 2047 and 3481 of *C. lindemuthianum* that overcome the most of known resistance genes in common bean. Furthermore, the *Co-Pa* gene in Paloma confers resistance to these and many other Mesoamerican races. On the other hand, the Mesoamerican genes *Co-4* and its alleles, *Co-5*, *Co-6* and other anthracnose Mesoamerican resistant genes confer effective resistance to Andean races that overcome the resistance of *Co-Pa* and other Andean resistance genes.

These results suggest that combining *Co-Pa* with Mesoamerican resistant genes in a single cultivar will confer effective and possibly durable resistance to all known races of the highly variable anthracnose pathogen of common bean. In addition, the flanking SNP markers SS82 and SS83 linked to resistance gene *Co-Pa* of Paloma, will be very useful for gene pyramiding of *Co-Pa* with other Mesoamerican and Andean genes.

## Conclusions

The results presented here showed that Paloma cultivar has a new dominant gene conferring resistance to anthracnose, which is independent from the *Co-1*, *Co-2*, *Co-3* ^*4*^, *Co-4, Co-4* ^*2*^ *, Co-4* ^*3*^ *, Co-5, Co-6, Co-12, Co-13, Co-14, Co-15* and *Co-16* genes, previously described. This gene, provisionally named as *Co-Pa* gene in Paloma, was mapped on the chromosome Pv01 of common bean at 390 kb region, flanked by SNP markers SS82 and SS83. The linkage between these markers and the *Co-Pa* gene will be extremely important for marker-assisted introgression of the gene into elite cultivars in order to enhance resistance.

## Additional files

Additional file 1: Table S1. Phenotypic evaluation of the 73 F_2:3_ families of the Paloma × PI 207262 cross inoculated with the *Colletotrichum lindemuthianum* race 3481 and genotyping of the corresponding F_2_ plants with nine KASP markers associated with the anthracnose resistance gene in Paloma. (XLSX 14 kb)

Additional file 2: Table S2. Phenotypic evaluation of the 90 F_2_ plants of the Paloma × PI 207262 cross inoculated with the *Colletotrichum lindemuthianum* race 3481 and genotyping with three KASP markers close linked to the anthracnose resistance gene in Paloma. (XLSX 13 kb)

## Abbreviations

ARS: Agricultural research service
BSA: Bulk segregant analysis
CNL: N-terminal coiled-coil domain
INTA: Instituto Nacional de Tecnología Agropecuaria
KASP: Kompetitive Allele Specific PCR
NB-LRR: Nucleotide-binding, leucine-rich repeat
Nupagri: Núcleo de Pesquisa Aplicada à Agricultura
PCR: Polymerase chain reaction
PI-PLC: Phosphoinositide-specific phospholipase-C
PTK: Protein tyrosine kinase
RB: Resistant bulk
SB: Susceptible bulk
SNP: Single nucleotide polymorphism
STK: Serine threonine protein kinase
TNL: N-terminal *Toll*-interleukin-1 receptor (TIR)-like domain
UEM: Universidade Estadual de Maringá
USDA: United States Department of Agriculture
